# First consumption of alcohol and drugs: When does it start, in which occasions and what can we do to prevent it?

**DOI:** 10.1016/j.pmedr.2025.103296

**Published:** 2025-11-04

**Authors:** Ivona Vrkić Boban, Marijan Saraga

**Affiliations:** a Department of Pediatrics, University Hospital of Split, Spinčićeva 1, Split, Croatia; b School of Medicine, University of Split, Šoltanska 2, Split, Croatia

**Keywords:** Alcohol, Drugs, First consumption, Circumstances, Children

## Abstract

**Objectives:**

The prevalence of alcohol use among adolescents in Croatia is high. The aim of this study was to determine the age and circumstances of first alcohol and drug consumption among adolescents.

**Methods:**

An online questionnaire survey was conducted among final-year high school students in Split-Dalmatia County, Croatia, between June 6 and December 28, 2020. Statistical analyses were performed using chi-square test, with a *P*-value <0.05 considered as statistically significant.

**Results:**

Among 1030 respondents, 92.33 % reported having consumed alcohol at least once, with 33 % initiating use at ≤14 years of age. The youngest age of the first alcohol consumption was three years, and the median age was 15 years (IQR = 14.00–16.00). Most participants first tasted beer (34.38 %), primarily out of curiosity (63.93 %), at their own home (28.29 %) friend's homes (27.44 %), or in nightclubs or café bars (27.23 %). A total of 24.27 % reported having tried drugs, most commonly marijuana (92.40 %), usually with friends (88.80 %). The youngest age of the first drug use was six years, and the median age was 16 years (IQR = 15.00–17.00).

**Conclusions:**

First use of alcohol and drugs occurs at an early age, often in familiar environments. Enhanced educational and preventive measures beginning in early childhood are essential to prevent engagement in risky behaviors among children.

## Highlights

• 92.33 % of high school students in Split-Dalmatia County, Croatia had at least once drank alcohol.
• 24.27 % of them have tasted drugs, in most cases marijuana.
• The lowest age of the first alcohol consumption was three years.
• The lowest age of the first drugs consumption was six years.
• Preventive and educational measures starting in the earliest childhood are needed.

## 1 Introduction

Alcohol consumption often begins during adolescence (Lees et al., 2020; Vrkić Boban and Saraga, 2018). However, some studies have reported that children may first taste alcohol between eight and 15 years of age (Aiken et al., 2018; Donovan and Molina, 2011; Gerónimo Carrillo et al., 2017), or even earlier (5–10 years) (Skylstad et al., 2022). Drinking at ≤14 years is considered early-onset drinking (Donovan and Molina, 2011). Drug use, most commonly marijuana, is usually initiated in late adolescence (Aly et al., 2020). Substance use at such an early age can interfere with normal development and has been linked to long-term physical, behavioral, social, and health risks (Aly et al., 2020; Lees et al., 2020). Marijuana use during adolescence has been associated with increased cortical thickness in the prefrontal regions (Albaugh et al., 2021), while alcohol consumption in early childhood can contribute to the development of substance dependence later in life (Aiken et al., 2018; Patrick et al., 2019).

Children begin to acquire knowledge about alcohol as early as age two, understand its role in adult culture, and develop certain alcohol-related expectancies by age four (Voogt et al., 2017). Alcohol expectancies, which begin to develop in early childhood, are important predictors of alcohol use later in life (Smit et al., 2019). Parental alcohol-specific attitudes and their educational measures (Bailly, 2016), as well as the frequency of alcohol use among parents, significantly influence children's early initiation of drinking (Donovan and Molina, 2014). Exposure to a father's alcohol use is more frequently associated with favorable alcohol expectancies among children (Smit et al., 2019). Growing up in a single-mother household (Donovan and Molina, 2011) or under less stringent parental supervision (Bailly, 2016; Donovan and Molina, 2011) can also lead to early initiation of drinking (Donovan and Molina, 2011). Living in a poor neighborhood environment increases the risk of early drinking initiation (Skylstad et al., 2022). In some less developed countries, children may begin drinking to cope with food insecurity or inadequate childcare or traumatic experiences (Skylstad et al., 2022).

In most cases, children first taste beer in their own homes (Gerónimo Carrillo et al., 2017; Jackson et al., 2015), often offered by a parent (Jackson et al., 2015), most commonly the father (Gerónimo Carrillo et al., 2017). Some mothers allow children to sip alcohol under their supervision at a young age, believing it may prevent later alcohol consumption during adolescence, when peers' influence is particularly strong (Jackson et al., 2012). Parenting education programs aimed at increasing parents' readiness to guide their children against early alcohol use could significantly reduce drinking at an early age (Ennett et al., 2016).

Children who begin drinking at a young age often have poorer school performance and strained relationships with parents (Bailly, 2016), are less religious, and more frequently engage in deviant behaviors (Bailly, 2016; Donovan and Molina, 2011). Early initiation of alcohol use has been linked to early alcohol intoxication (Morean et al., 2018; Patrick et al., 2019), alcohol-related problems (Aiken et al., 2018; Bailly, 2016; Patrick et al., 2019) and adult alcohol dependence (Guttmannova et al., 2011). Children who use cannabis before the age of 16 have an increased risk of mental health problems and substance use disorders later in life (Aly et al., 2020; ESPAD Group, 2020).

The ESPAD (European School Survey Project on Alcohol and Other Drugs) is a collaborative effort of independent research teams that collects data on substance use among 15–16-year-old students in more than 40 European countries. In the 2019 report, 90 % of respondents in Croatia had tried alcoholic beverages in their lifetime, while 21 % reported having tried marijuana (ESPAD Group, 2020). Among them, 58 % had consumed alcohol in the past 30 days (ESPAD Group, 2020). Croatia ranks among the countries with the highest prevalence of alcohol use among adolescents (ninth place) and heavy episodic drinking (five or more drinks on one occasion), following Denmark, Germany, Austria, Slovakia, and Georgia (ESPAD Group, 2020). Additionally, 9.2 % of Croatian respondents reported marijuana use in the past 30 days, placing the country twelfth among European countries (ESPAD Group, 2020). In Croatia, alcohol is most frequently consumed during festive occasions, and some adults occasionally offer children a sip of alcohol for fun. Independent alcohol use among children most often begins in social settings with peers, typically during high school or late elementary school years. In Croatia, drinking and selling alcohol is prohibited for individuals under 18 years of age, similar to some other countries (Aiken et al., 2018).

To our knowledge, no studies have examined the age and circumstances of first alcohol and drug consumption among children in Croatia. Therefore, we conducted a large study among final-year high school students in Split-Dalmatia County (SDC) to assess the prevalence and circumstances of early substance use, with the aim of informing timely planning and implementation of preventive measures for children and their parents.

## 2 Methods

### 2.1 Setting and participants

The study was conducted in SDC, the largest county in Croatia with approximately 423,407 inhabitants (Croatian Bureau of Statistics [Internet], 2024). It formed part of a larger research project among final-year high school students in SDC designed to examine the prevalence, circumstances and risk factors for risky behaviors (Vrkić Boban and Saraga, 2022; Vrkić Boban and Saraga, 2025). The Croatian educational system includes primary (elementary) (ages 6–13) and secondary (high) school (ages 14–18). In SDC, there are 49 high schools; 9 gymnasiums, 29 vocational schools and 11 mixed schools offering both gymnasium and vocational programs. Gymnasiums are general secondary schools that provide broad knowledge and prepare students for further education at colleges and universities. Vocational schools focus on acquiring professional knowledge, skills and competencies required for specific occupations. The duration of gymnasium programs is four, while vocational programs last three to four years. In the 2020/2021 academic year, 17,227 students attended high schools in SDC. To achieve the largest possible response and the most reliable results, we included all 49 high schools in the county. Previous studies have shown that the prevalence of substance use is highest among senior high school students (Vrkić Boban and Saraga, 2018). Therefore, only final-year high students were included in this study (3rd or 4th grade, depending on the school program) in age > 16 and < 20 years. In total, 4347 final-year high school students were enrolled across the 49 high schools in SDC in the 2020/2021 academic year.

### 2.2 Survey development

We developed a self-administered questionnaire using Google Forms*.* The survey contained 74 questions covering participants' characteristics (e.g., socioeconomic status, family structure, place of residence, school performance, religious practices, and parents' education) and their patterns of risky behaviors (frequency and circumstances of drug, alcohol, and tobacco consumption and negative consequences of alcohol consumption), as well as the frequency and characteristics of alcohol consumption during the COVID-19 lockdown. The survey consisted of information about the research and its objectives and informed the participants that completing the questionnaire would be considered as providing informed consent.

Before data collection, we contacted principals and pedagogues of all 49 high schools in SDC (via email or phone), informed them about the research, and sent them a sample of the questionnaire. After receiving signed informed consent to perform the survey, we provided a network link, which they forwarded to students (through e-classrooms, school websites, and similar platforms). Only students who voluntarily agreed to participate in the study accessed the survey. Participation was completely voluntary and anonymous, and no personal identifying data were collected. To achieve a higher response rate and more valid results, we organized a prize game in which one random participant was awarded with 1000 HRK (Croatian kuna;€133). Anonymity was maintained in a way that respondents who wanted to participate in the prize game had to add a secret password at the end of the survey questionnaire. Each student could access the survey only once. Only surveys with valid and consistent answers through the questionnaire were included, and those who reported places of attendance out of the SCD were excluded from the research.

### 2.3 Data collection

Data were collected from June 6 to July 7, 2020, and from October 12 to December 28, 2020. During this period, 1263 students completed the survey (response rate = 29.05 % of all final-year high school students in SDC). Of these, 1030 met the inclusion criteria. A total of 42 of the 49 high schools in SDC (85.71 %) participated in the study.

### 2.4 Statistical methods

Statistical variables were presented as the median and interquartile range, and categorical variables were presented as absolute numbers and percentages. The chi-square test was used to compare groups. Associations were assessed between gender groups; between students who had consumed alcohol at least once and those who had never consumed it; and between those who had consumed drugs and those who had never consumed them. Among participants who had consumed drugs or alcohol, associations were further assessed between different category variables for every answer (type of drinks: beer, mixed drinks, shots, wine, cocktail, champagne, other; place of first alcohol consumption: in their own home, at a friend's home, in a nightclub or café/bar, in open spaces, other; the reason for the first alcohol consumption: out of curiosity, out of boredom, to feel better, not to be the only one who is not drinking, festive occasions, emotional reasons, to be “cool,” parental provision of alcohol, for fun, due to persuasion of peers, other; frequency of drinking: every day, 1–2 times per week, 3–6 times per week, 2–3 times per month, ≤1 time per month, 1–3 times per year; 4–11 times per year; type of drugs consumed: marijuana, cocaine, amphetamines (speed), multiple different drugs, dimethyltryptamine, ecstasy, all mentioned drugs, other; frequency of drug consumption: once, 2–4 times in a lifetime, 5–10 times, >10 times, every time when going out with friends, occasionally when going out with friends, every day; who do they consume drugs with: alone, with family members, with a boyfriend or girlfriend, with friends, other). A *P-*value <0.05 was considered as statistically significant. Statistical analyses were conducted using SPSS version 25 (IBM, Armonk, New York; 2017).

### 2.5 Ethical approval

The Ethics Committee of the Faculty of Medicine in Split approved the study (Class: 003–08/20–03/0005, Ed. No.: 2181–198–03-04-20-0069). All procedures were conducted in accordance with the Declaration of Helsinki.

## 3 Results

A total of 1263 final-year high school students completed the questionnaire survey. Of these, 1030 met the inclusion criteria, comprising 433 boys (42.04 %) and 597 girls (57.96 %); *P* < 0.01. The median age was 18 years (IQR = 17.00–18.00).

Most respondents (*N* = 951; 92.33 %; *P* < 0.01) reported consuming alcohol at least once in their lifetime (Table 1). Among them, 872 (84.66 %) reported current alcohol use, most frequently 2–3 times per month (Table 1). No significant gender differences were observed in alcohol consumption (*P* = 0.33).

**Table 1:** Prevalence and circumstances of the first alcohol consumption among final year high school students (N = 1030) in Split-Dalmatia County, Croatia; data were collected between June 6 and December 28, 2020.

		N	%	*P*⁎
Ever consumed alcohol	Yes	951	92.33	<0.01
	No	79	7.67	
Type of consumed alcoholic drink	Beer	327	34.38	<0.01
	Mixed drinks	285	29.97	
	Shots	158	16.61	
	Wine	128	13.46	
	Cocktails	47	4.94	
	Champaign	2	0.22	
	Other	4	0.42	
Place of first alcohol consumption	In their own home	269	28.29	<0.01
	At friend's home	261	27.44	
	In nightclub or cafe bar	259	27.23	
	In open spaces	147	15.46	
	Other	15	1.58	
The reason for first alcohol consumption	Out of curiosity	608	63.93	<0.01
	Out of boredom	102	10.73	
	To feel better	79	8.31	
	Not to be the only one who isn't drinking	44	4.63	
	Other	32	3.35	
	Festive occasions	24	2.52	
	For emotional reasons	19	2.00	
	To be “cool”	17	1.79	
	Because parent gave me alcohol	13	1.37	
	Due to the peers´ persuasion	9	0.95	
	For fun	4	0.42	
Frequency of drinking	Every day	17	1.95	<0.01
	1–2 times per week	122	13.99	
	3–6 times per week	38	4.36	
	≤ 1 time per month	179	20.53	
	2–3 times per month	214	24.54	
	1–3 times per year	143	16.40	
	4–11 times per year	159	18.23	

⁎ χ2 test.

**Fig. 1:**
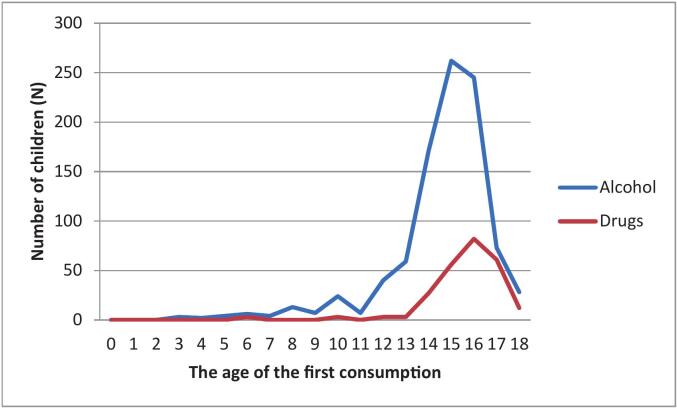
Distribution of the age of the first alcohol and drugs consumption among final year high school students (*N* = 1030) in Split-Dalmatia County, Croatia; data were collected between June 6 and December 28, 2020.

The youngest reported age of the first alcohol consumption was three years, with a median of 15 years (IQR = 14.00–16.00). Students most commonly reported their first alcohol consumption at age 15 (*N* = 262; 27.55 %) or 16 (*N* = 245; 25.44 %) (Fig. 1).

In most cases, the first consummated alcoholic drink was beer (34.38 %), followed by mixed drinks (29.97 %) and shots (16.61 %); *P* < 0.01 (Table 1).

Students were most often drinking alcohol for the first-time in their own homes (28.29 %). Other popular places for the first alcohol consumption were friends' homes (27.44 %), nightclubs and café bars (27.23 %), or open spaces such as beaches, parks, etc. (15.46 %, *P* < 0.01),Table 1.

The most commonly reported reason for first alcohol use was curiosity (63.93 %), followed by boredom (10.73 %), and to feel better (8.31 %); *P* < 0.01, Table 1.

Nearly one-quarter of respondents (24.27 %); *P* < 0.01 reported drug use at least once in their lifetime. Among boys, 28.18 % reported lifetime drug use, compared with 21.44 % of girls (*P* = 0.01).

The median age of first drug consumption was 16 years (IQR = 15.00–17.00), while the youngest reported age was six years. Most of the final-year high school students (32.80 %) reported first using drugs at age 16 (Fig. 1).

The vast majority of participants (92.40 %) had tasted marijuana; *P* < 0.01, (Table 2).

**Table 2:** The prevalence and circumstances of the first drugs consumption among final-year high school students (N = 1030) in Split-Dalmatia County, Croatia; data were collected between June 6 and December 28, 2020.

		N	%	*P*⁎
Ever consumed drugs	Yes	250	24.27	<0.01
	No	780	75.73	
Type of consumed drugs	Marijuana	231	92.40	<0.01
	Cocaine	5	2.00	
	Speed	5	2.00	
	More different types	3	1.20	
	Dimethyltryptamine	2	0.80	
	Other	2	0.80	
	Ecstasy	1	0.40	
	All above drugs	1	0.40	
Frequency of drugs consumption	Once	31	12.40	<0.01
	2–4 times in lifetime	50	20.00	
	5–10 times	50	20.00	
	>10 times	50	20.00	
	Every time when going out with friends	2	0.80	
	Occasionally when going out with friends	40	16.00	
	Every day	27	10.80	
Who do they consume drugs with	Alone	8	3.20	<0.01
	With family members	6	2.40	
	With boyfriends or girlfriends	8	3.20	
	With friends	222	88.80	
	Other	6	2.40	

⁎ χ2 test.

The same amounts of respondents (20 %) reported consuming drugs 2–4 times in their lifetime, 5–10 times and more than 10 times, while 10.80 % consume drugs every day; *P* < 0.01, (Table 2).

Drugs were most commonly used with friends (88.80 %). A smaller proportion reported using drugs alone (3.20 %) or with family members (2.40 %), (Table 2).

## 4 Discussion

We found that a large proportion of high school students in SDC reported lifetime alcohol and drug use, with initiation occurring at an early age. These findings highlight the need for planning and implementing preventive measures beginning in early childhood to prevent the risk of heavy substance use among children and adolescents.

The vast majority of final-year high school students in SDC (92.33 %) reported alcohol use at least once in their lifetime. This prevalence is higher than that reported in the ESPAD 2019 survey for European respondents overall (79 %) (ESPAD Group, 2020) and in other studies (Heradstveit et al., 2017), but similar to the ESPAD 2019 report for prevalence of alcohol consumption among Croatian students (90 %) (ESPAD Group, 2020). Such high prevalence of drinking among high school students in SDC is concerning because alcohol use in childhood and adolescence can interfere with brain and pubertal development, contribute to various diseases, and increase the risk of alcohol dependence in adulthood (Aly et al., 2020; Bailly, 2016; Lees et al., 2020; Patrick et al., 2019).

The youngest reported age of first alcohol consumption among respondents was three years. This could have resulted from accidental consumption or maybe a consequence of child neglect or abuse. Alcohol drinking in Croatia is a socially accepted behavior, and society has a high tolerance for drinking alcohol as an accepted style of behavior deeply rooted in tradition (Mišević et al., 2020). Alcohol is commonly consumed in small quantities with meals (Mišević et al., 2020) or at festive occasions such as family celebrations and weddings, during which parents or family members sometimes offer a sip for fun. These results indicate that parents and all social members should be cautioned that such behaviors are unacceptable because even small amounts of alcohol at an early age can have long-term consequences (Aly et al., 2020; Lees et al., 2020). Preventive programs beginning in early childhood and aimed at educating parents about all negative consequences of such early consumption may help reduce early drinking initiation among children.

The median age of first alcohol consumption among respondents was 15 years and 33 % reported first tasting alcohol at age ≤ 14 years. Bailly reported that 60–70 % of French 11-year-olds had tasted alcohol (Bailly, 2016), Donovan et al. reported that 25 % of children in Pennsylvania, United States had tasted alcohol by age ≤ 14 years (Donovan and Molina, 2011) while Mares et al. reported that 40 % of Dutch 12-year olds had tried alcohol (Mares et al., 2011). Morean et al. reported that children in Connecticut usually tasted their first alcoholic drink at a mean age of 11.32 years and began drinking alcohol at age of 14.04 years (Morean et al., 2018). In our study, most respondents first tasted alcohol at ages 15 and 16. These findings suggest that implementing educational programs about negative consequences of alcohol use during childhood—particularly in the final years of elementary and first years of high school—together with providing interesting extracurricular activities, may help reduce early alcohol initiation among children.

Students in SDC most often drank beer (34.38 %), consistent with findings from some studies (Gerónimo Carrillo et al., 2017), while others reported that children first tasted wine (Donovan and Molina, 2008). Alcohol was usually first consumed in students' own homes (28.29 %), as previously reported (Donovan and Molina, 2008; Jackson et al., 2015). Similar proportions reported drinking alcohol in their friend's homes (27.44 %) or in nightclubs and café bars (27.23 %). These results indicate that children should be under better adult supervision while socializing at home to prevent alcohol consumption among them. Although Croatian law prohibits the sale and serving of alcohol to those younger than 18 and bans drinking in public places, 85.33 % of the final-year high school students in SDC reported being able to purchase alcohol in markets or nightclubs and café bars without difficulty (Vrkić Boban and Saraga, 2025). Drinking in public places can result in a fine of €150. Stricter enforcement measures—such as more frequent police patrols in nightclubs, café bars, and public places; higher penalties for establishments that sell alcohol to children and adolescents; and higher penalties for parents whose children are found drinking alcohol in public places—could help reduce underage alcohol consumption.

Curiosity was the most frequently reported reason for first-time alcohol consumption among our respondents (63.93 %), followed by boredom (10.73 %) and the desire“ to feel better”(8.31 %). Kuntsche et al. reported that 42.50 % of their respondents drank alcohol first in their lifetime “to make a toast,” 36.40 % to see how it would affect them, and 31 % to have more fun (Kuntsche and Müller, 2012). Boys were more likely to drink out of curiosity, whereas girls more often reported drinking to cope (Kuntsche and Müller, 2012). Early exposure to alcohol advertisements in social media (e.g., during every football game beer advertising appears on television) or alcohol consumption among peers may foster curiosity, which in turn becomes a common trigger for initial alcohol consumption among children.

Nearly one-quarter of our respondents (24.27 %) reported having tried drugs at least once in their lifetime, and 10.80 % reported daily use. These results indicate a higher prevalence of drug use among students in SDC than reported in some studies (ESPAD Group, 2020; Heradstveit et al., 2017; Tahiraj et al., 2016), but lower than others (Malatestinić et al., 2005).

The youngest reported age of first drug consumption was six years, with a median age of 16 years. Sartor et al. found that marijuana consumption starts at the age of 11 years (Sartor et al., 2019), while Kingston et al. reported that 42 % of children start consuming alcohol, nicotine, or marijuana at an early age, with 31 % of them ≤11 years (Kingston et al., 2018). Drug use at such an early age may indicate child neglect or abuse and also reflects the easy availability of drugs to children and young people in SDC. Better social and preventive measures among risky families (e.g., those in which family members consume drugs) and patrols around schools to prevent selling drugs to children could help reduce drug consumption among children and adolescents in SDC.

Students in SDC have most often reported consuming marijuana (92.40 %) with their friends (88.80 %), consistent with findings from some other studies (ESPAD Group, 2020; Malatestinić et al., 2005). Peer pressure is one of the most common reasons to start and continue using substances, as well as having a parent or family member with substance use disorder (Jackson et al., 2015). Strict parenting, religious influences, hanging out with friends who do not use psychoactive substances (Kuntsche and Müller, 2012), good relationships and communication with mothers, and mothers' negative attitudes among early-onset drinking (Ennett et al., 2013) could prevent substance use among children and adolescents (Kuntsche and Müller, 2012).

Our results indicate that alcohol use often begins at an early age. Communication with children about negative effects of alcohol consumption from the earliest age, as well as parental education about implementation of preventive measures in early childhood, may help prevent early drinking initiation (Ennett et al., 2013; Mares et al., 2011). Early preventive measures could prevent alcohol and drug use in later life, especially because some studies showed that among several children acute alcohol intoxication occurred one year after their first alcohol consumption (Jackson et al., 2015; Patrick et al., 2019). Others reported that first drunkenness usually occurs 3.32 years after first alcohol tasting (Morean et al., 2018). Earlier age of the first alcohol consumption, first drinking outside the house, and shorter interval between the age of the first alcohol consumption and first alcohol intoxication significantly increase the risk for heavy drinking, binge drinking, and development of other alcohol-related problems in adolescence (Aiken et al., 2018; Patrick et al., 2019). Parents' restrictive attitudes toward underage drinking are one of the strongest protective factors for risky drinking behaviors among children, and adolescents whose parents enforce strict alcohol-specific rules are less likely to engage in risky drinking behaviors (Agabio et al., 2015). Frequent and open alcohol-specific communication between parents and adolescents increases their perceptions of the negative consequences associated with alcohol use and can reduce adolescents' alcohol consumption (Agabio et al., 2015), as can effective parental monitoring (Agabio et al., 2015).

Besides parental educational programs, school-based alcohol interventions across multiple individual and group sessions could also reduce early alcohol use among children (Stigler et al., 2011). These programs are made to enhance students' knowledge about alcohol and skills to help them resist pressure to use alcohol (Stigler et al., 2011). Using interactive teaching techniques (e.g., small-group activities and role plays), they address social and environmental risk factors with special attention on adolescents who are particularly at risk, helping students identify and resist social influences (e.g., by peers and media) to use alcohol (Stigler et al., 2011).

In Croatia, several different educational preventive programs have been constructed, but only some of them were implemented (Croatian Institute of Public Health, 2025). In Zagreb County, a preventive program among adolescents who have risk factors for substance use and their family members was developed, but it is still not performed (Croatian Institute of Public Health, 2025). The aim was to prevent addiction through developing attachment to school, strengthening social skills, resilience to peer pressure, and family support by working with parents, and teachers, and educating professionals to ensure continuous and comprehensive support through group and individual workshops, online counseling, and street art (Croatian Institute of Public Health, 2025). A similar program was performed in Osijek, named “Alphabet of the prevention” among elementary school children with the aim of promoting youth health protection and adopting healthy lifestyles, focusing on socially acceptable forms of behavior and reducing interest in addictive substances, and proper use of free time through various extracurricular and after-school activities (Croatian Institute of Public Health, 2025). It included work with students, teachers, and parents with the aim that children develop their self-esteem and a positive self-image, cooperative social skills, and non-violent conflict resolution skills (Croatian Institute of Public Health, 2025). In Primorsko-Goranska County, some interesting lectures about negative consequences of drug use have been held for first year elementary school students (Croatian Institute of Public Health, 2025). Similar programs have been implemented in SDC to empower adolescents with behavioral problems and their families through psychosocial support, improving their knowledge and skills related to experimenting with addictive substances, organizing peer support as a form of prevention of risky behaviors, affirming and promoting healthy lifestyles, and educating and raising public awareness about addictive behaviors of young people by promoting protective factors (Croatian Institute of Public Health, 2025). The mental health service of the Public Health Institute of the SDC also runs some preventive projects in schools and makes telephone, individual, or group counseling to promote mental health and prevent substance use (Croatian Institute of Public Health, 2025).

With parents, teachers, and family members, pediatricians also have an important role in the prevention of substance use among children (Kulig, 2005). They should discuss substance use in their routine healthcare with children and their parents, starting from an early age, try to identify children at risk for substance use, and provide intervention and treatment measures if necessary (Kulig, 2005). Avoiding exposure to substance-related content on television and in movies, limitations on alcohol advertising, and implementing education programs in classrooms or social media can also help to prevent substance use among children and adolescents (Stacy et al., 2004; Strasburger, 2010).

### 4.1 Limitations and strengths of the study

The study was designed as a questionnaire survey, so the findings rely on the sincerity of the participant's responses. However, because the survey was anonymous, we believe that students provided honest answers. The study was conducted online due to the COVID-19 lockdown period when schools were closed. Only final-year high school students were included, yet the study is large, representing 29.05 % of all final-year high school students in SDC, and is the first study to assess the prevalence and circumstances of early alcohol and drug use in SDC. Although the results cannot be generalized, they indicate potential harmful behaviors among children and adolescents in Croatia. Alcohol and drug consumption at such an early age can have multiple long-term consequences on health (Aiken et al., 2018; Aly et al., 2020; Bailly, 2016; Donovan and Molina, 2014; Lees et al., 2020), emphasizing the importance of collaboration among parents, schools, and the entire community to delay initial alcohol and drug consumption as much as possible. Notably, alcohol consumption among adolescents in Croatia is very high, possibly due to early age initiation.

## 5 Conclusion

The prevalence of alcohol and drug consumption among high school students in SDC is high and often begins at an early age. Initiation and implementation of educational and preventive measures among parents, children, teachers, pediatricians, and social media from early childhood could help reduce substance use among children and adolescents and mitigate its long-term negative consequences.

## CRediT authorship contribution statement

**Ivona Vrkić Boban:** Writing – original draft, Validation, Project administration, Investigation, Formal analysis, Data curation, Conceptualization. **Marijan Saraga:** Writing – review & editing, Supervision, Project administration, Conceptualization.

## Ethical statement

The Ethics Committee of the Faculty of Medicine in Split approved the research among students (Class: 003–08/20–03/0005, Ed. No.: 2181–198–03-04-20-0069) and the procedures were in accordance with the Helsinki Declaration.

## Declaration of competing interest

The authors declare that they have no known competing financial interests or personal relationships that could have appeared to influence the work reported in this paper.

## Data Availability

Data will be made available on request.
